# Pan-cancer analysis of transcriptional metabolic dysregulation using The Cancer Genome Atlas

**DOI:** 10.1038/s41467-018-07232-8

**Published:** 2018-12-14

**Authors:** S. R. Rosario, M. D. Long, H. C. Affronti, A. M. Rowsam, K. H. Eng, D. J. Smiraglia

**Affiliations:** 1 Department of Cancer Genetics and Genomics, Roswell Park Cancer Institute, Buffalo, 14263 NY USA; 2 Department of Biostatistics, Roswell Park Cancer Institute, Buffalo, 14263 NY USA

## Abstract

Understanding metabolic dysregulation in different disease settings is vital for the safe and effective incorporation of metabolism-targeted therapeutics in the clinic. Here, using transcriptomic data for 10,704 tumor and normal samples from The Cancer Genome Atlas, across 26 disease sites, we present a novel bioinformatics pipeline that distinguishes tumor from normal tissues, based on differential gene expression for 114 metabolic pathways. We confirm pathway dysregulation in separate patient populations, demonstrating the robustness of our approach. Bootstrapping simulations were then applied to assess the biological significance of these alterations. We provide distinct examples of the types of analysis that can be accomplished with this tool to understand cancer specific metabolic dysregulation, highlighting novel pathways of interest, and patterns of metabolic flux, in both common and rare disease sites. Further, we show that Master Metabolic Transcriptional Regulators explain why metabolic differences exist, can segregate patient populations, and predict responders to different metabolism-targeted therapeutics.

## Abstract (web summary)

Metabolism is frequently dysregulated in cancer. Here, the authors conduct a pan-cancer analysis of The Cancer Genome Atlas to determine significant metabolic pathway alterations, highlight master metabolic transcriptional regulators, and predict patient response to metabolism targeted-therapeutics

## Introduction

Despite waning interest in how metabolism influences cancer, recent efforts have brought renewed awareness of cancer as a metabolic disorder^1–3^. While the field was first introduced to cancer as a glycolytic disease, often described as the Warburg effect^4^, modern advancements have pointed to other metabolic dependencies, such as fatty acid metabolism in prostate cancer (PRAD)^5^. These investigations have led to the inclusion of metabolic reprogramming as a new hallmark of malignant transformation^6^. However, the extent to which all metabolic genes and pathways are expressed by cancers of different origins, and how they differ from one another, is largely underexplored. Even more pressingly, how these metabolic pathways and genes differ from non-malignant, normal human tissues have yet to be determined. Few existing papers attempt to explain differences between cancer and normal tissues that can be leveraged to understand metabolic reprogramming, based on genomic perturbation^7–10^. While some innate differences between tumor and normal tissues are addressed^7^, the focus is typically on common mechanisms and metabolic gene dysregulation that exist in pan-cancer, rather than those changes that exist in an individual tumor type dysregulation, and how these affect existing treatment^9,10^ and chemosensitivity^11^. Others look at this concept solely from the metabolomic angle, in a single disease site^8^. These studies, while informative, create a gap in which we question if there are targetable metabolic pathways unique to a single disease site and whether there is a way to distinguish patients who will respond to these metabolic-targeted therapies.

Scientific consortiums like The Cancer Genome Atlas (TCGA)^12^ encourage comprehensive genomics approaches in large numbers of patients with many different cancer types, as well as their matched normal tissues. This transcriptomic data has already been used to explore and explain a wide variety of important questions of cancer biology, particularly those aimed at understanding immune response in different disease states^13^ and oncogenic drivers^14,15^. For example, determining new ways to classify tumors, whether by their cell of origin and immunophenotype^16^ or their clinical outcome endpoints^17^, are helping to assign treatments and treatment usage, especially in chemoresistant patients^11^. Others have looked more specifically at known oncogenic molecular processes and pathways to better explain how genomic mutations can impact expression and signaling^14,15^, highlighting combination therapy potential^15^. These studies point to the utility of using transcriptomic data to exploit biologically relevant vulnerabilities, but do not focus on metabolic-targeted therapies. Our approach, instead, focuses explicitly on using transcriptomic data to identify metabolic vulnerabilities for 114 different pathways.

TCGA data allows the opportunity to address whether metabolic genes differ between normal and malignant conditions across diverse tissues of origin. While metabolomics, the systemic study of small molecules utilized and left behind during essential cellular processes^18^, is the most comprehensive way to understand the metabolic composition of cells at a given time, the technique is still in its infancy^19^. Conversely, abundant and readily available transcriptomic data exists for large numbers of patients in many types of cancer. Such datasets provide the opportunity to investigate the variety of mechanisms cancers utilize to control metabolic enzyme expression to achieve metabolic reprogramming, including feedback and crosstalk between metabolite pools and transcription^20^.

Recently, transcriptomics data in conjunction with current biochemical understanding have been exploited to construct genome-scale metabolic workflows^21^. This has been especially successful in *Escherichia coli*, in which over half of the metabolic outputs from >450 different reactions within the organism were correctly modeled^22,23^. Nevertheless, extrapolating metabolic changes from transcriptomics is not without its challenges, as stoichiometric relationships and kinetic information must be assumed. However, a recent study provided convincing evidence for the extrapolation of metabolite levels from transcriptomic data, based on high levels of significant correlation between the two in a detailed look at breast cancer RNA-sequencing and unbiased metabolomics^24^.

An additional challenge to understanding cancer metabolic reprogramming lies in determining the genetic and epigenetic changes controlling metabolic phenotypes. To this end, we suggest elucidating expression and alteration of master metabolic transcriptional regulators (MMTRs) may provide novel understanding of why metabolism differs in varying tissues. Ohno^25^ first recognized master transcriptional regulators, using the term to describe transcription factors (TFs) that regulate sets of genes which determine developmental fate. Master regulators (MRs) have been implicated in a variety of disease states^26,27^ and with several genomic alterations^28,29^. More recently, MRs have become interesting as biomarkers of disease^30,31^ and pharmacological targets^32^.

However, a more nuanced understanding of unique metabolic dependencies, or weaknesses, in specific cancer types, subtypes, or even tissues of origin, may provide novel mechanisms for therapeutic targeting with lower toxicity than traditional chemotherapeutics. A recent example is the recognition that cancers that are deficient in the methylthioadenosine phosphorylase enzyme are highly susceptible to inhibition of methionine adenosyl transferase 2A (MAT2A), resulting in reduced function of protein methyltransferase 5^33^.

Metabolic therapies provide an attractive approach in the clinic, due to evasion of both single and multi-drug resistance in tumors, thus far^34^. Determining responders to these metabolic therapies, however, has proven challenging. There are currently no studies determining the MMTRs of specific metabolic pathways, which may serve as drivers of metabolic phenotypes. These would also provide insights into ways to therapeutically leverage metabolic dependencies and segregate patient populations by predicted response to metabolic-targeted therapeutics.

Therefore, the aim of this study was to comprehensively assess which metabolic pathways have altered metabolic transcriptional profiles in 26 different cancer types as compared to their matched normal tissues. Here we demonstrate that we have the ability to not only segregate different disease sites and different molecular subtypes of the same disease, but also to predict response to metabolism-targeted therapy. This selective drug sensitivity is further explained by individual pathway MMTRs. This represents a means of identifying a mechanism by which these metabolic pathways become distorted in malignancy and offers novel targets for intervention.

## Results

### Pan-cancer screen for transcriptional metabolic dysregulation

**Fig. 1:**
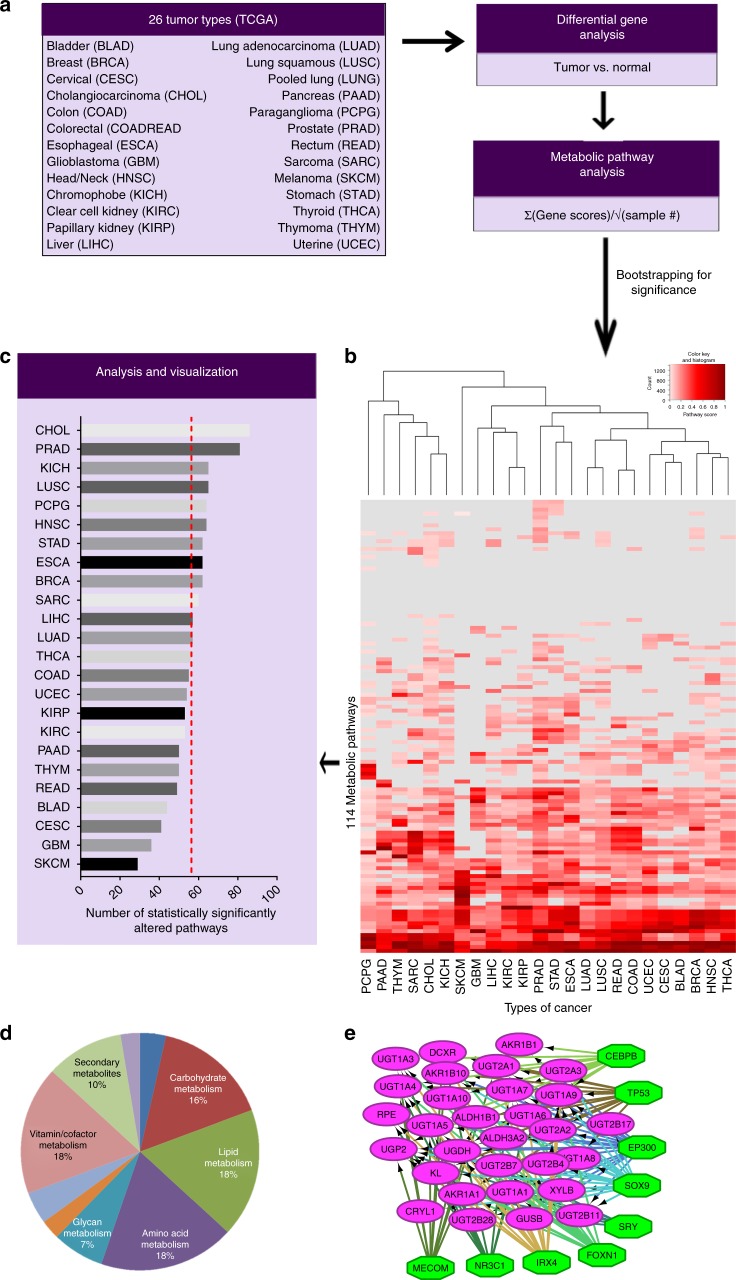
Transcriptional metabolic pathway analysis methods pipeline. **a** Twenty-six cohorts of tumor samples, including two pooled sets (COADREAD and LUNG) from The Cancer Genome Atlas (TCGA), with matched normal samples, were utilized to determine the transcriptional metabolic profiles specific to each type of cancer, as compared to their normal. **b** Pathway scores ((Σlog FC* − log(adj.*p*.val))/√*n*), for 114 metabolic pathways from KEGG, were then calculated based on the results of differential expressed gene (DEG) analysis using Limma to compare tumors to matched normal. Pathways are then bootstrapped for significance, to determine which pathways are highly dysregulated as compared to chance. Those pathways are then plotted in a heatmap, with the type of cancer as the *x*-axis and the 114 pathways as the *y*-axis. Non-significant pathways are gray and a gradient from white to red for those pathways significantly dysregulated and the intensity of red indicating the magnitude of dysregulation. **c** The number of significant pathway scores are then summed to determine which types of cancers are most metabolically dysregulated at the transcriptional level, as compared to the average number of dysregulated pathways (dashed line). **d** The pathways were then sorted into each of the 10 major metabolic pathway subtypes defined by KEGG and later underwent **e** master regulator analysis via iRegulon

To screen for transcriptional metabolic dysregulation, we used TCGA^12^ RNA-sequencing data from 26 different types of cancer with matched normal samples (Fig. 1a, Supplementary Data 1) applying a custom analysis pipeline (Fig. 1). Magnitude of metabolic dysregulation was calculated by determining DEGs, which includes log fold changes and adjusted *p* values, comparing tumor with normal matched samples and assigning scores based on 114 metabolic pathways from The Kyoto Encyclopedia of Genes and Genomes (KEGG)^35^. Adjusted *p* value magnitude is affected by sample size, which varies across datasets. To account for how such variation would affect the metabolic pathway score, each was divided by the square root of *n* (the sample size) for each disease site. Bootstrapping methods were then used to determine which pathways were significantly (red) or non-significantly (gray) dysregulated (Fig. 1b). The metabolic dysregulation scores (Fig. 1c) were then confirmed in separate patient populations for prostate^36^, lung adenocarcinoma (LUAD)^37^, and breast carcinoma^38^ (Supplementary Figure 1). Pathway scores in validation cohorts were significantly correlated with those from TCGA cohorts with the same disease, demonstrating the pipeline’s robustness. To determine patterns of metabolic dysregulation, individual pathway scores were segregated into ten major metabolic classes (Fig. 1d). Additionally, MMTRs were determined for individual pathways, such as the pentose glucuronate interconversion (PGI) pathway (Fig. 1e), to elucidate drivers of unique metabolic phenotypes existing in cancers of different origins.

**Fig. 2:**
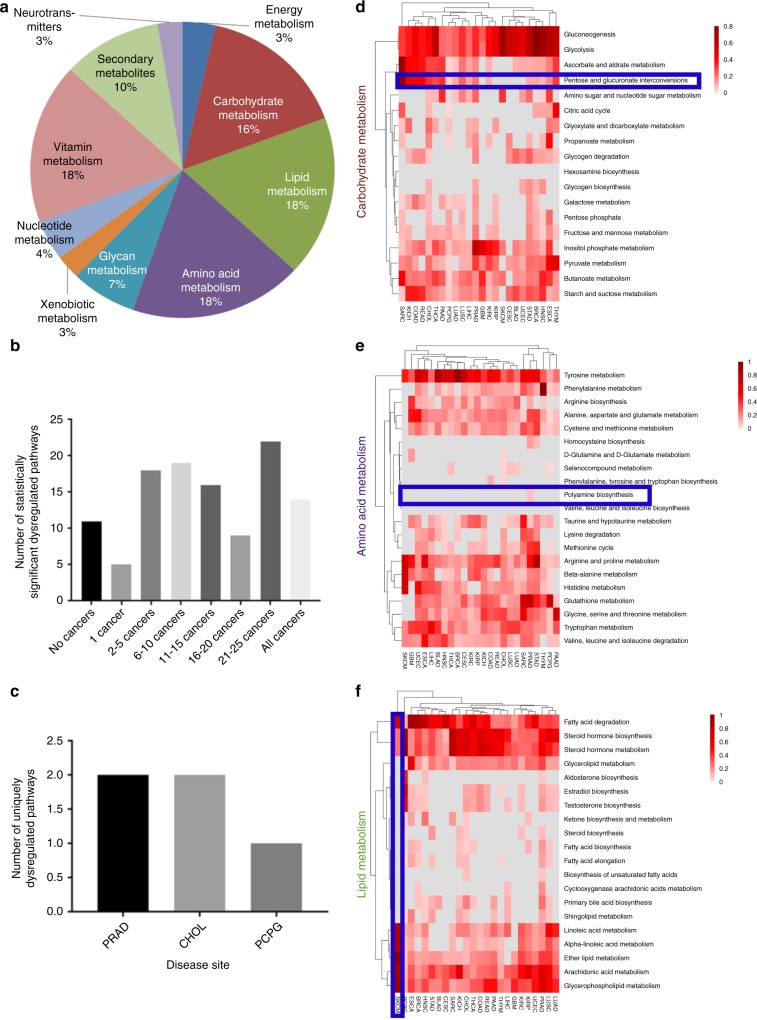
Ascertaining pathways of interest by looking at major types of metabolic pathways. **a** One hundred and fourteen metabolic KEGG pathways broke down into 10 major metabolic types of pathways. This allowed for the identification of **b** pathways that were statistically significantly altered in a specific number and variety of cancers and **c** the number of uniquely dysregulated pathways in specific tumor types. Each of these major metabolic categories was then broken down into individual heatmaps of bootstrapped pathway scores where gray are non-significant altered pathways and the gradient of red represents the magnitude of dysregulation (combination of both up-dysregulation and down-dysregulation) in each of the pathways across the cancer cohorts. **d** In carbohydrate metabolism, a pathway largely altered across all tumor types, like pentose and glucuronate interconversions, is highlighted. **e** Within amino acid metabolism, the polyamine biosynthetic pathway is highlighted as an example of cancer-type-specific dysregulation. **f** Subcutaneous melanoma (SKCM) was identified as the cancer type with the highest degree of dysregulation, based on Euclidian distance, within a subset of the KEGG pathways in the lipid metabolism category

The 114 individual metabolic pathways were condensed into ten major metabolic categories based on KEGG classifications (Fig. 2a). After bootstrapping, pathways in each classification were then further broken down by the number of cancers for which they were dysregulated (Fig. 2b). Additionally, we identified unique pathways, altered in one disease site (Fig. 2c, Supplementary Data 6). For example, PRAD had two pathways uniquely dysregulated (polyamine biosynthesis and nicotinamide adenine dinucleotide biosynthesis). Analysis of pathways within major metabolic categories revealed patterns of dysregulation reflective of common metabolic reprogramming in cancer, like patterns consistent with the Warburg effect, such as dysregulation of glycolysis (Fig. 2d), to varying degrees in all cancer types. While these results are in agreement with the literature, the method also discovered additional metabolic disruption across the 26 cancers examined.

One such finding is the common, but not universal, dysregulation of pentose and glucuronate interconversion (Fig. 2d), which is significantly altered in 20 cancer types. Due to its high degree of dysregulation, this pathway has been studied in some cancer types, including liver hepatocellular carcinoma (LIHC)^39,40^. However, little is known about this pathway in sarcoma (SARC), where we find it most strongly dysregulated. Meanwhile in only six types of cancer PGI was not significantly dysregulated. Conversely, some pathways were dysregulated in a single type of cancer, like polyamine biosynthesis in PRAD, suggesting unique dependencies of certain cancers (Fig. 2e).

Within specific metabolic categories, hierarchical clustering highlighted disease sites with distinct levels of dysregulation. For example, lipid metabolism disruption in skin cutaneous melanoma (SKCM), whose level and pattern of dysregulation within this category caused SKCM to segregate separately from others (Fig. 2f). SKCM is one of the least metabolically dysregulated cancers, with 30 of 114 pathways significantly dysregulated, much less than the mean number of dysregulated pathways, 58 of 114 (Fig. 1c).

### Pathways dysregulated across multiple cancer subtypes

**Fig. 3:**
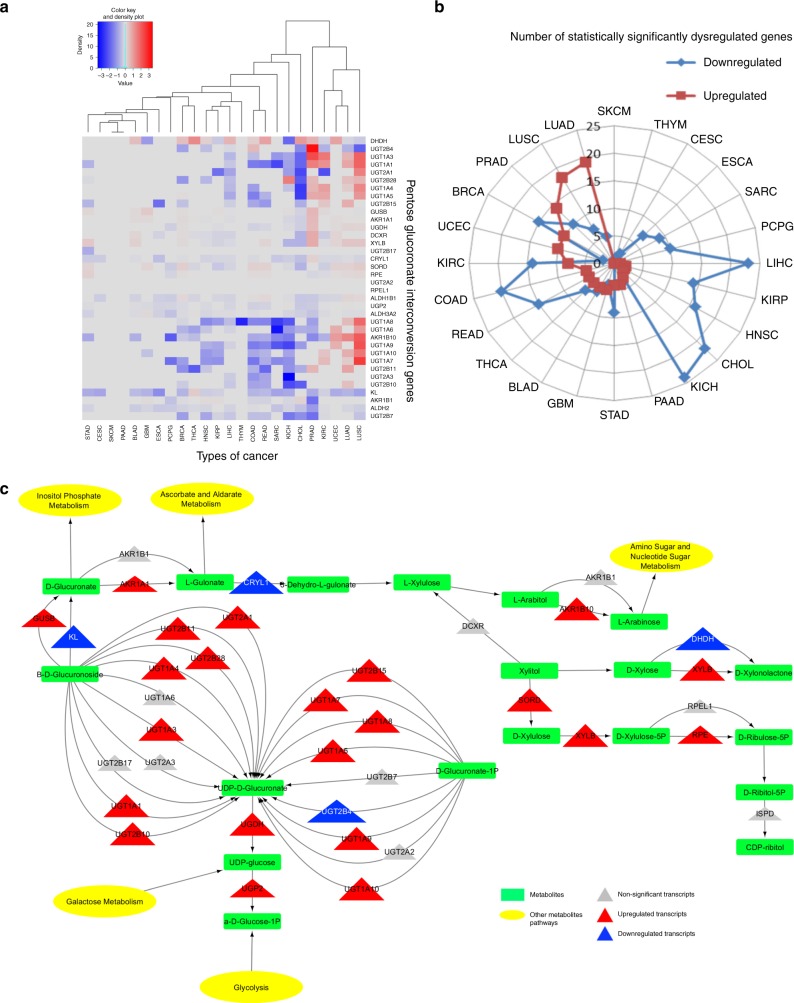
The pentose glucuronate interconversion pathway is dysregulated across all types of cancer, but to a different extent and in different directions. **a** Heatmap of fold changes, showing the differences in direction of transcriptional change in tumor over normal for each individual gene within the pentose glucuronate interconversion pathway, across all cancer types. **b** Radar plot demonstrating the number of statistically significant up-regulated (red) and down-regulated (blue) genes within a pathway, for each of the indicated cancers. **c** Model of the pentose glucuronate interconversion pathway in LUAD, which has the largest number of statistically significant up-regulated genes. Pathway displays genes (triangles) shaded by direction (red and blue, up and down, respectively) and significance and sized by fold-change differences. Pathways also include metabolite outputs (green rectangles) and connected pathways (yellow ellipse)

The PGI pathway clusters closely with glycolysis and gluconeogenesis, which are significantly dysregulated in all cancer types (Fig. 2b). This pathway is involved in interconversions of monosaccharide pentose and glucuronate, the salts or esters of glucuronic acid. While it is known that this pathway is frequently dysregulated in hepatocellular carcinoma, little is known about the dysregulation of this pathway in the context of other disease sites^40,41^. As is shown in the carbohydrate metabolism heatmap, many cancer types highly dysregulate PGI, while only a few do not (Fig. 2b). To better understand expression changes within PGI, the 35 individual genes of this pathway were examined in detail across cancer types (Fig. 3a–b). This approach demonstrated two distinct groups of cancer sites, those significantly up-regulating and those down-regulating a majority of genes within this pathway.

Consistent with what has previously been reported in LIHC, our analysis found this disease site is amongst the cancers with the most significantly dysregulated genes, nearly all of which are down-regulated (23 of 35). Additionally, we obsevered that SARC, kidney chromophobe (KICH), cholangiocarcinoma (CHOL), and colon adenocarcinoma also have a high degree of down-regulation within the PGI pathway, but the magnitude of down-regulation exceeds LIHC. Conversely, the lung cancer subtypes, LUAD and lung squamous cell carcinoma (LUSC), up-regulate the greatest number of genes within the pathway, though the magnitude of up-regulation is far greater in LUSC. While it has been reported that intermediate metabolites of PGI are increased in LUAD^42^, the transcriptional up-regulation of 20 of the 35 genes within this pathway are unknown. Previous literature has also pointed to the dysregulation of this pathway in LUSC, but failed to further explore how this pathway was transcriptionally disrupted^43^.

Modeling the metabolic pathways by placing significantly dysregulated genes within the metabolic circuit may predict which metabolites will be most readily affected and in what direction. For example, comparing PGI models in LUAD (Fig. 3c) and LIHC (Supplementary Figure 2) reveal two different metabolic pictures. A large number of genes within the pathway that are up-regulated in LUAD, but down-regulated in LIHC, contribute to the generation of UDP-d-glucuronate from β-d-glucuronoside and d-glucuronate-1 phosphate. Therefore, the expression levels of these enzymes predicts for relatively high UDP-d-glucuronate levels in LUAD, but low levels in LIHC. This finding is highly consistent with previous metabolomic studies in LUAD, which have asserted increased UDP-d-glucuronate levels, in cancer tissues, as compared to matched normal^42^, as well as the literature regarding global PGI metabolite down-regulation in LIHC^41^.

### Pathways uniquely dysregulated within a single cancer subtype

**Fig. 4:**
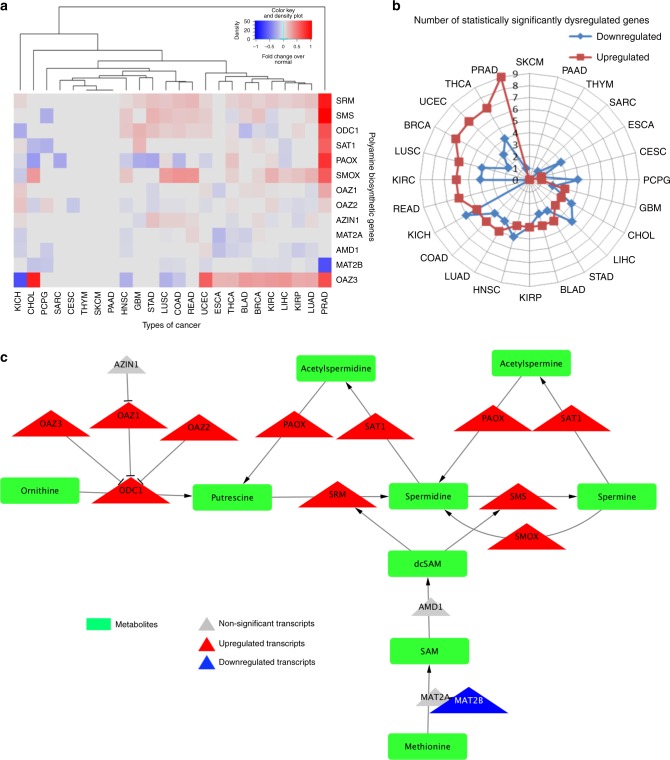
The polyamine biosynthetic pathway is highly specifically dysregulated in prostate cancer. **a** Heatmap of fold changes, showing the differences in direction of transcriptional change in tumor over normal for each individual gene within the polyamine biosynthetic pathway, across all cancer types. **b** Radar plot demonstrating the number of statistically significant up-regulated (red) and down-regulated (blue) genes within a pathway, for each of the indicated cancers. **c** Model of the polyamine biosynthetic pathway in PRAD, which has the largest number of statistically significant up-regulated genes. Pathway displays genes (triangles) shaded by direction (red and blue, up and down, respectively) and significance and sized by fold-change differences. Pathways also include metabolite outputs (green rectangles)

This metabolic pipeline can also elucidate pathways uniquely dysregulated in specific cancer types. An example of this is the unique polyamine disruption in PRAD (Figs. 2e, 4a). Polyamines are small, positively charged molecules with many functions, impacting almost every aspect of cell survival^44^. While this pathway is important in every cancer, the PRAD-unique dysregulation of this pathway is particularly interesting because flux through the biosynthetic pathway is extremely high in normal prostate, due to the high rate of acetylated polyamine secretion into the prostatic lumen^45^. Not only does PRAD have the highest number of significantly up-regulated (compared to normal prostate) genes in this pathway (Fig. 4b), they also show the greatest magnitude of change, which is reflected in the large Euclidean distance observed by unsupervised hierarchical clustering (Fig. 4a).

Modeling of the polyamine metabolic circuit clearly demonstrates an increase in polyamine biosynthesis and catabolism in PRAD (Fig. 4c). This is reflected in increased expression of both rate-limiting enzymes in the pathway (*ODC1* and *AMD1*), and significant increases in catabolic enzymes *SAT1* (spermidine/spermine *N*1-acetyltransferase 1), *PAOX*, and *SMOX*. The pathway is further enhanced by *MAT2A* up-regulation and down-regulation of the inhibitory subunit *MAT2B*, predicting for enhanced SAM production feeding into *AMD1* as well as greatly increased expression of the polyamine synthases, *SRM* and *SMS*. These findings are consistent with well-documented increased level of polyamines, acetylated polyamines, and other metabolites within polyamine biosynthesis in PRAD, all of which can be predicted by the modeling approach^46,47^. Conversely, in KICH, the rate-limiting enzymes *ODC1* and *AMD1* are significantly down-regulated, suggesting reduced levels of polyamines (Fig. 4a, Supplementary Figure 3). Additionally, there is an increase in *SAT1*, which increases acetylation of polyamines, but a decrease in *PAOX*, which decreases back-conversion of acetylated polyamines to un-acetylated polyamines. These transcriptional changes, taken together, lead to polyamine depletion in cancer, as compared to normal tissues. This broad up-regulation of polyamine biosynthesis in PRAD suggests a unique dependence on its function, as compared to other disease sites, providing rationale for pharmacological intervention.

**Fig. 5:**
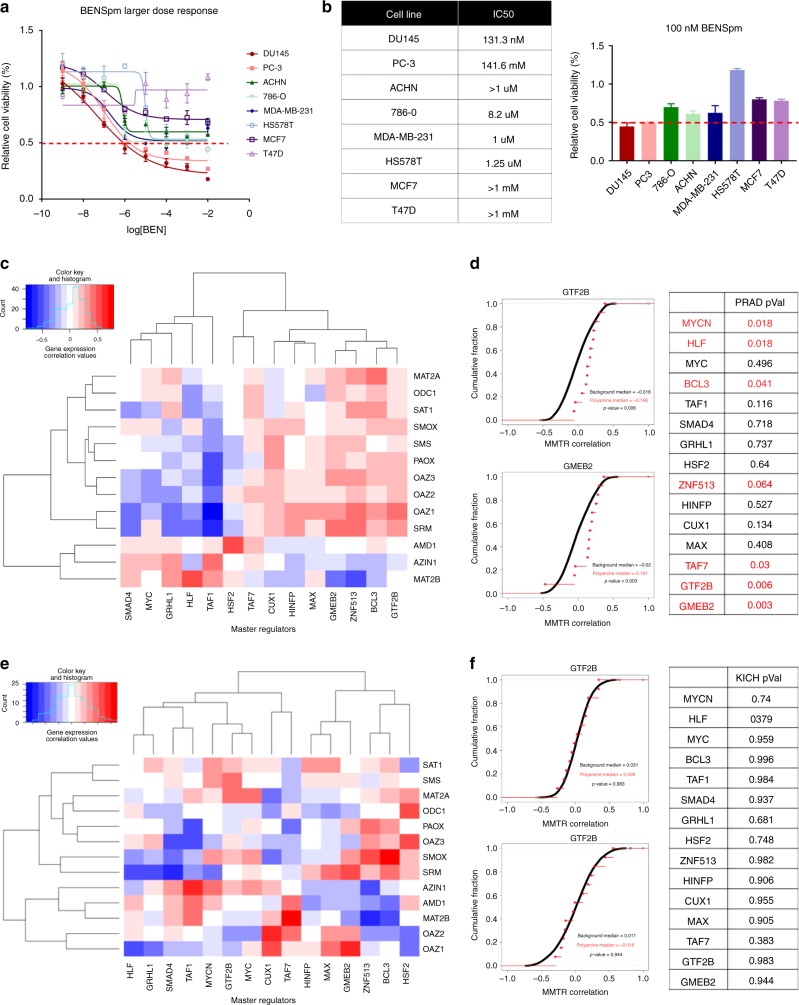
Targeting polyamine biosynthesis is highly effective in prostate cancer, and master metabolic transcription regulators (MMTRs) may explain why **a** dose–response curves show prostate cancer cell lines (PC-3 and DU145) that are most sensitive to SAT1 activation by BENSpm, as compared to kidney cancer cell lines (ACHN and 786-O) and breast cancer cell lines (MCF7, MDA-MB-231, and HS578T) (*n* = 3). **b** This is confirmed by calculation of IC_50_s. **c** Prostate cancer (PRAD) heatmap of correlations between MMTRs (*x*-axis) and the polyamine biosynthetic genes (*y*-axis) with the intensity representing strength of correlation (blue is negative, red is positive) and **d** cumulative distribution frequencies showing MMTR correlation with every gene in the genome, with red dots indicating the correlations with polyamine biosynthetic genes, show a distinct pattern and high level of statistically significant correlation values between MMTRs and polyamine biosynthetic genes. *P* values for all MMTRs and polyamine genes are reported in the table, with significant associations highlighted in red. **e** Kidney chromophobe (KICH) heatmap of correlations between MMTRs (*x*-axis) and the polyamine biosynthetic genes (*y*-axis), with the intensity representing strength of correlation (blue is negative, red is positive) and **f** cumulative distribution frequencies showing MMTR correlation with every gene in the genome, with red dots indicating the correlations with polyamine biosynthetic genes, show an random pattern and non-statistically significant correlation values between MMTRs and polyamine biosynthetic genes. *P* values for all MMTRs and polyamine genes are reported in the table, with significant associations highlighted in red. *Error bars = s.d

The drug N^1^, N^11^-bis(ethyl)norspermine (BENSpm), a SAT1 stabilizer that increases polyamine acetylation, was utilized to understand whether prostate cancer cell lines could more selectively be targeted by further destabilization of polyamine metabolism. When comparing the sensitivity of eight cell lines, two prostate cancer (DU145 and PC-3), two kidney cancer (ACHN and 786-O), and four breast cancer lines (MDA-MB-231, HS578T, T47D, and MCF7) (Fig. 5a–b), the prostate cancer lines were most sensitive to BENSpm treatment.

These differences in polyamine biosynthesis dysregulation between cancer types may be explained by MMTRs. When analyzing the overall pathway scores, the two most highly dysregulated cancer types are PRAD and KICH (Fig. 4a). As previously demonstrated, when directionality is taken into account, these pathways are largely dysregulated in opposite directions (Fig. 4a). Utilizing iRegulon^48^, which pairs motifs and chromatin immunoprecipitation-sequencing (ChIP-seq) tracks to determine TFs controlling the gene networks, a list of MMTRs of polyamine biosynthesis was constructed (Supplementary Figure 4). We then correlated MMTR expression with the expression of individual genes in the polyamine biosynthetic pathway in both PRAD and KICH cohorts (Fig. 5c). Distinct patterns of correlation emerged in PRAD, where a majority of the MMTRs were significantly correlated, either positively or negatively, with polyamine biosynthetic genes. Comparisons of the cumulative distribution frequencies of correlations between MMTRs and all genes in the genome (black) with correlations of these MMTRs with polyamine biosynthetic genes only (red) show that polyamine gene correlations are statistically significant when considering global expression patterns, demonstrated by a shift in the distributions (Fig. 5d). Conversely, KICH lacks a strong pattern of correlation between MMTRs and polyamine biosynthetic genes (Fig. 5e, Supplementary Figure 5). This finding was confirmed by cumulative distribution analysis, where there was no significant relationship between MMTRs and polyamine biosynthetic gene expression observed when considering global transcriptional patterns (Fig. 5f). The top four MMTRs (BCL3, GMEB2, GTF2B, and ZNF513), with the strongest collective positive correlation, are important for polyamine biosynthetic gene regulation, as evidenced by an iRegulon^48^ network, which demonstrates that these four MMTRs are collectively predicted to regulate 10/13 of the genes from the pathway (Supplementary Figure 6). Thus, the metabolic analysis pipeline can discover targets of pharmacologic intervention. Further, this information combined with MMTR analysis can be a useful tool providing insights into drivers of metabolic reprogramming across and within cancer sites.

### BRCA subtype metabolic reprogramming

**Fig. 6:**
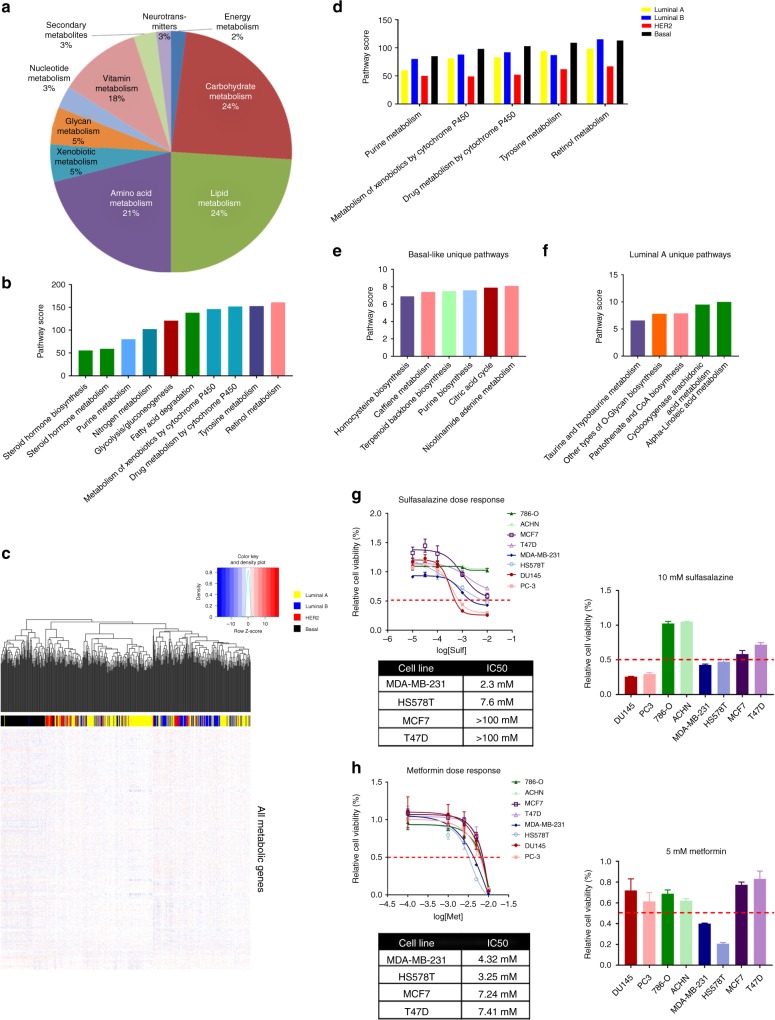
Metabolic dysregulation distinguishes BRCA molecular subtypes and defines therapeutic sensitivity. **a** Breakdown of 62 significantly dysregulated metabolic pathways in the BRCA pooled data. There is a roughly equal dysregulation of amino acid, carbohydrate, and lipid pathways. **b** The top pathways dysregulated in BRCA come from a wide variety of major categories, like nucleotides and lipids. However, the two most dysregulated pathways in BRCA are a vitamin-associated pathway, retinol metabolism, and an amino acid pathway, tyrosine metabolism. **c** Unsupervised clustering of BRCA patients, who were classified based upon the PAM50, on all metabolic genes, reveals a tight cluster of the basal-like subtype (black), which are highly metabolically dysregulated as explained by the magnitude of dysregulation, as displayed in the heatmap. While luminal A (yellow), luminal B (blue), and HER2-expressing (red), did not cluster as tightly, there are still recognizable groups of these patients. **d** The Top 5 pathways that overlapped between all four subtypes of patients are shown here. While these pathways are highly dysregulated in all four subtypes, they vary to different extents and are almost always highest in the basal-like cells. **e** After metabolic pathway scoring, pathways unique to the basal-like, and (**f**) luminal A patients were plotted. The top 5 unique pathways for each of the subtypes are shown. **g** Targeting the homocysteine biosynthetic pathway with sulfasalazine reveals increased sensitivity in basal-like cells, as compared to luminal A cells, as emphasized by the IC_50_ values (*n* = 3). **h** Targeting the citric acid cycle with metformin reveals increased sensitivity in basal-like cells, as compared to luminal A cells, as emphasized by the IC_50_ values (*n* = 3). *Error bars = s.d.

BRCA is one of the most metabolically dysregulated cancer types, in terms of the sum of pathway scores (Fig. 1c). Analysis of all BRCA cases reveals a large level of dysregulation of carbohydrate, lipid, and amino acid metabolism in roughly equal proportions (Fig. 6a). Additionally, the top dysregulated pathways seem to be encompassing pathways from each major category (Fig. 6b). Importantly, BRCA consists of four molecular subtypes with distinct treatments and outcomes for patients: luminal A, luminal B, HER2, and basal^49^. This, therefore, leads us to question whether these four major subtypes had distinct transcriptional metabolic profiles.

Using the PAM50^50,51^, a set of 50 DEGs utilized to classify BRCA, all patients were assigned to one of the four subtypes. Patients were first randomly clustered based on the expression of all metabolic genes (Fig. 6c). Basal-like tumors (black), clustered very distinctly from the luminal A (yellow), luminal B (blue), and HER2-expressing (red) counterparts, indicating a strong metabolic shift in these patients. While not as distinct, smaller clusters did form for each of the other molecular subtypes. Each molecular subtype was then compared to the normal tissue, and DEG lists for each independent subtype were utilized to determine which of the 114 pathways were significantly dysregulated. This analysis revealed a total of 89 dysregulated pathways, some of which were missed entirely by an analysis of BRCA pooled data. Pathways like tyrosine metabolism and retinol metabolism, as well as glycolysis and gluconeogenesis, are dysregulated across all subtypes, but to a different degree (Fig. 6d), as indicated by different scores for each pathway among the four molecular subtypes. While many pathways were dysregulated across all subtypes, there were distinct pathways present in each molecular subtype. For example, in basal-like tumors, the most aggressive forms of BRCA, terpenoid backbone biosynthesis, homocysteine biosynthesis, and the citric acid cycle (CAC), are uniquely dysregulated, amongst others (Fig. 6e). Meanwhile, in luminal A tumors, the subtypes with the most favorable prognosis, α-linoleic acid metabolism, taurine and hypotaurine metabolism, and cyclooxygenase arachidonic acid metabolism, were uniquely dysregulated (Fig. 6f). Further, this unique metabolic pathway dysregulation in molecular breast subtypes suggests potential differential sensitivity to therapeutic intervention.

Since homocysteine biosynthesis is specifically dysregulated in the basal-like subtype, we utilized the drug sulfasalazine, an Xc cysteine-glutamate transport inhibitor that decreases intracellular homocysteine pools^52^, to understand whether basal breast cancer cell lines could more selectively be targeted by homocysteine cycle destabilization. When comparing the sensitivity of eight cell lines, two prostate cancer (DU145 and PC-3), two kidney cancer (ACHN and 786-O), two luminal breast cancer (MCF7 and T47D), and two basal breast cancer (MDA-MB-231 and HS578T) (Fig. 6h), we did see increased sensitivity of basal breast cancer to sulfasalazine, as compared to the luminal breast cancer cells. Interestingly, prostate cancer cells were more sensitive, which is a finding consistent with the fact that our analysis identifies PRAD as one of two disease sites with significantly dysregulated homocysteine biosynthesis (Fig. 2e). It is also notable that this pathway was not detected as dysregulated in the BRCA cohort, but is dysregulated specifically in the basal-like subtype.

Additionally, the drug metformin, a first-generation biguanide, principally thought to be a mitochondrial complex 1 inhibitor, has been shown to decrease glucose metabolic flux through the CAC^53^. Therefore, metformin was utilized to determine whether basal breast cancer cells could more selectively be targeted, due to the unique dysregulation of the CAC. When comparing the sensitivity of eight cell lines, two prostate cancer (DU145 and PC-3), two kidney cancer (ACHN and 786-O), two luminal breast cancer (MCF7 and T47D), and two basal breast cancer (MDA-MB-231 and HS578T) (Fig. 6h), we did see increased sensitivity of basal breast cancer to metformin, as compared to any other cells.

**Fig. 7:**
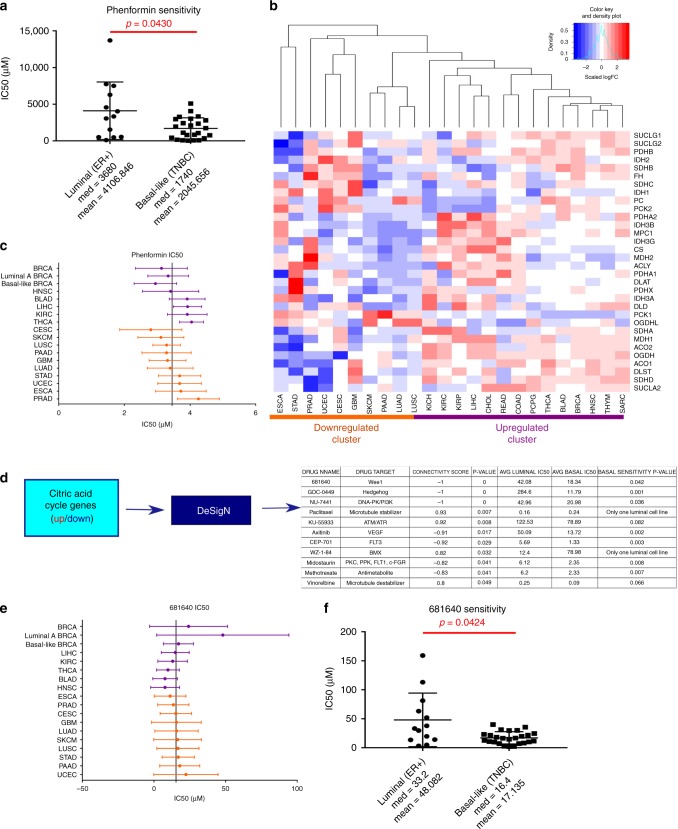
Basal-like BRCA cells are more sensitive to citric acid cycle targeting than luminal-like BRCA cells. **a** The Sanger Database of Genomics of Drug Sensitivity in Cancer shows a statistically significant difference in sensitivity to phenformin between basal and luminal-like breast cancer cell lines. **b** Hierarchical clustering of all TCGA cancer cohorts revealed two major groups of citric acid cycle gene expression: highly up-regulated (purple) and highly down-regulated (orange). **c** Analysis of sensitivity to phenformin in cell lines of different cancer types comparing those with citric acid cycle gene up-regulation (purple) and down-regulation (orange). **d** DeSigN was then used based on the citric acid cycle signature to determine drugs that would preferentially target cells with CAC dysregulation and identified 11 drugs, most of which had significant differences in drug sensitivity between luminal and basal breast cell lines. **e** Data downloaded for cell lines with TCGA classifications that were utilized in this analysis revealed those cancer types with increased numbers of up-regulated genes (purple) were in fact more sensitive (lie to the left of the mean IC_50_ line) than those with more down-regulated genes (orange). **f** There is a statistically significant difference in the IC_50_s between basal breast cancer cell lines and luminal cell lines, indicating that basal cell lines are more sensitive, as predicted. *Error bars represent s.d.

This was further confirmed using the Sanger Genomics in Drug Sensitivity Database^54^, where data were downloaded for phenformin, a second-generation biguanide. In this larger drug screen, which included 13 luminal breast cancer and 24 basal breast cancer cell lines, the basal cells demonstrated a statistically significant 2-fold increase in sensitivity to phenformin (Fig. 7a), confirming the sensitivity to metformin drug treatments. Clustering was then performed for all TCGA disease sites for the CAC genes and identified a distinct signature for two groups of cancers: those that preferentially up-regulate or down-regulate CAC genes (Fig. 7b). Sensitivity to phenformin across all cohorts of TCGA-associated cell lines were plotted (Fig. 7c), and demonstrated that although basal breast cancer cells are indeed more sensitive, the cancer types with up-regulated pathways were not more sensitive than down-regulated cancer types. Due to the lack of clear trends in phenformin response, the DeSigN database^55^ was used to determine additional drugs whose efficacy would depend on CAC gene dysregulation. Multiple drugs were predicted (Fig. 7d) to have a stronger effects on basal breast cancer, which highly dysregulates the CAC. This is shown by statistically significant increased sensitivity in a majority of basal, as compared to the luminal, cell lines. One drug with a high connectivity score, 681640 (wee-1 inhibitor), was utilized to better understand sensitivity across multiple disease types. As compared to the mean half-maximal inhibitory concentration (IC_50_) across all cell lines, those with TCGA classifications, that fell within the up-regulated group (Fig. 7e, purple) were more sensitive than those within the down-regulated group (Fig. 7e, orange), with the exception of BRCA which showed increased variability. This variability is most likely due to statistically significant differences in IC_50_s between basal and luminal subtypes, where basal subtypes were more sensitive, as predicted (Fig. 7e, f). Overall, it is noteworthy that magnitude of distortion of a metabolic pathway can accurately predict drug sensitivity, even when those drugs do not explicitly target the metabolic pathway.

**Fig. 8:**
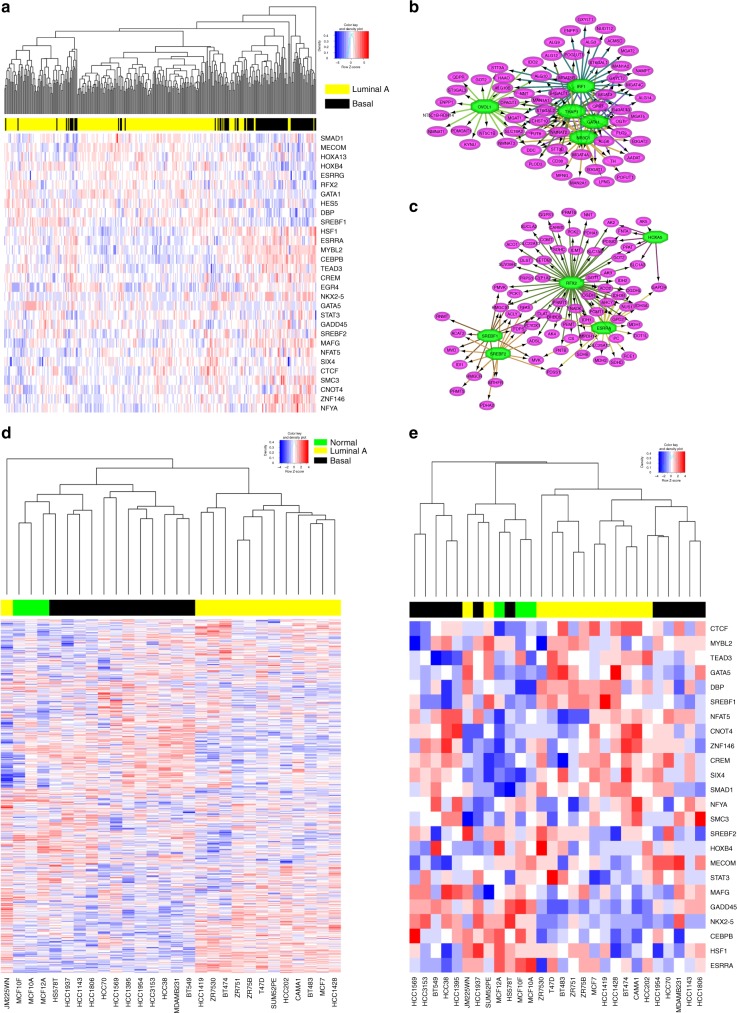
Master metabolic transcriptional regulators (MMTRs) distinguish BRCA molecular subtypes, and BRCA cell lines. **a** Unsupervised clustering of luminal A (yellow) and basal-like (black) patients on the expression levels of all MMTRs of their unique pathways (listed in Fig 6e and f) create separate clusters of patients. **b** MMTR analysis of the pathways unique to basal-like patients revealed a network of 14 MMTRs, the five most highly enriched are shown here. **c** MMTR analysis of the pathways unique to luminal A patients revealed a network of 15 MMTRs, the five most highly enriched are shown here. **d** Unsupervised clustering of 28 cell lines representing normal breast (n = 3) (green), basal breast cancer (n = 12) (black), and luminal breast cancer (n = 13) (yellow) on all metabolic genes reveals a tight cluster of each of the distinct cell line types. **e** Unsupervised clustering of normal (green), luminal (yellow), and basal-like (black) cell lines on the expression levels of all MMTRs of their unique pathways create separate clusters

Given the differential therapeutic sensitivity based on specific pathway distortion, we asked what drives this distortion in patient populations. MMTRs of uniquely dysregulated pathways (Fig. 8a) in luminal A (Fig. 8b) and basal (Fig. 8c) subtypes of BRCA were identified. The top 5 MMTRs in basal-like unique pathways (SREBF1, ESRRG, ESRRA, RFX2, and SREBF2) differ from those associated with the luminal A unique pathways (IRF8, OVOL1, THAP1, GATA1, and TFAP2C). Further, MMTR expression levels cluster patients based on their molecularly defined BRCA subtypes, where basal (black) patients are independent from luminal A (yellow) patients (Fig. 8a). The distinct metabolic profiles and ability of MMTRs to accurately distinguish normal breast cells from luminal A and basal breast cells were further confirmed using RNA-sequencing data from 27 different cell lines^56^. First, with the exception of one luminal cell line (JM225CWM), the metabolic gene expression clearly segregated the luminal, basal, and normal breast cell lines (Fig. 8d). Second, when we clustered cell lines on MMTR expression levels, defined in Fig. 8a, cell lines segregated by molecular subtype. These MMTRs provide possible explanations for differences in metabolic reprogramming between different BRCA subtypes.

## Discussion

The present study applies an analytical pipeline utilizing transcriptomic information to characterize changes in metabolic pathways associated with cancer. The approach successfully profiled metabolic reprogramming in 26 cancer types, revealing common and unique patterns of disruption in major cancer cohorts, as well as metabolic vulnerabilities distinguishing molecular subtypes within the same disease site.

The scoring algorithm yields insights that cannot be acquired through DEG analysis alone by looking not only at the magnitude of changes occurring in a particular gene, but also how meaningful that change is to the disease site. Combining the fold change and adjusted *p* value into a single score allows scaling of the importance of gene expression changes within a metabolic pathway. The bootstrapping approach then accounts for changes in a way that identifies patterns not expected simply by chance (Supplementary Figure 7). This is an important aspect of our pipeline, as it accounts for the varying degree of tumor-associated transcriptional drift across cancer types, as well as tissue procurement error and/or contaminating cell types associated with cohort samples at tissue-specific rates. Furthermore, mapping these genes onto metabolic circuits, with magnitude of change and direction (up-regulation or down-regulation), allows for determination of patterns indicating convergence of effect on key metabolites.

High degrees of correlation between population pools from different transcriptomic platforms (RNA-sequencing/microarrays) further demonstrate the robustness of this approach (Supplementary Figure 1). These additional datasets implicate many of the same pathways as being highly dysregulated in PRAD^36^, LUAD^37^, and BRCA^38^ as compared to normal matched tissues. Confirmation in three separate patient populations, on different transcriptomic platforms, reveals biologically relevant metabolic pathway dysregulation, and that our scoring approach is highly robust.

Two related studies have addressed metabolic patterns of disease through transcriptomic analysis, successfully identifying metabolic differences either between normal and cancer^7^ or across cancers^24^. We expand upon this by looking at expression difference of MMTRs for specific metabolic pathways and their correlation with magnitude of metabolic pathway dysregulation (Figs. 5, 8). MMTRs provide putative mechanistic insight into observed metabolic profiles and are associated with target gene expression. They can be genetic drivers and are clinically relevant molecular signatures in cancer cohorts. Combining identification of drug sensitivity and correlation with degree of pathway dysregulation (Fig. 7) allows for target patient population identification (Fig. 8).

An important test of validity for the metabolic pathway scores generated is modeling metabolic circuits, predicting expected metabolite pool alterations, and comparing that with published metabolomic studies. The PGI pathway is well known to be dysregulated and almost exclusively studied in LIHC^39,40^. However, we suggest that there are cancers dysregulating this pathway to a greater degree, including LUAD, where there is significant pathway-specific gene up-regulation (Fig. 3). Unbiased metabolomic studies comparing LUAD with normal lung tissue identified significant UDP-d-glucuronate elevation^42^. As shown in Fig. 3c, the significant gene expression changes in LUAD patient samples would be expected to divert metabolites towards the UDP-d-glucuronate production, which connects to several other pathways^57,58^. Interestingly, LUSC up-regulates this pathway more than LUAD. However, little metabolomic data exists in the context of this disease site. Nevertheless, our metabolic pathway scores implicate this pathway as being at least equally important in LUSC.

Perhaps not surprisingly, we also found metabolic pathways with highly restricted patterns of dysregulation like polyamine metabolism in prostate cancer (Fig. 4a). It is well established that this pathway is highly active in normal prostate, and further enhanced in prostate cancer^45,46^. The nearly complete biosynthetic and catabolic enzymes up-regulation in PRAD is striking, and the idea of increased metabolic flux is supported by metabolomics^47^. Additionally, therapeutic targeting of polyamine biosynthesis with BENSpm is highly effective and selective, highlighting the utility of the metabolic pipeline in determining metabolic pathways of interest for pharmacologic intervention.

Further, we identified a set of MMTRs for genes in polyamine biosynthesis whose expression highly and positively correlated with significant up-regulation of those genes in PRAD. In contrast, polyamine biosynthesis is down-regulated in KICH and exhibited weaker, non-significant correlations between MMTR expression and genes within the pathway. Additionally, MMTR association with common cancer-type-specific mutations may indicate differences in metabolic reprogramming for specific patient populations based upon co-occurrence or mutual exclusivity. For example, TMPRSS2-ERG fusion in PRAD, one of the most frequently occurring mutations, is mutually exclusive with GTF2B overexpression, a highly enriched MMTR. Interestingly, there is a significant amount of overlap amongst ERG and GTF2B-binding sites, and in PRAD ChIP-seq data, ERG peaks have been identified in three polyamine genes, potentially explaining their mutual exclusion (Supplementary Figure 4). This highlights the potential for different genetic drivers of disease to cooperate with altered MMTR expression to drive specific patterns of metabolic reprogramming.

Also of interest was the identification of patient subsets within breast cancer exhibiting unique patterns of metabolic reprogramming. Understanding metabolic profiles of different molecular subtypes are important in disease sites like BRCA, where different subtypes have distinct treatment regimens and outcomes (Fig. 6). The basal-like subtype, also known as triple-negative breast cancer, as defined by the PAM50^50,51^ clusters tightly based on metabolic genes and exhibits more highly dysregulated metabolic pathways (Fig. 6c). We effectively exploited this metabolic difference using drugs targeting homocysteine biosynthesis (Fig. 6g), as well as those that either target the CAC (Fig. 6h) or those predicted to be more effective when the CAC is dysregulated (Fig. 7). In both cases, basal-like cell lines are more sensitive than luminal A-like cell lines, in agreement with the fact that these pathways were uniquely dysregulated in the basal-like patients. The identification of MMTRs driving the differences in metabolic dysregulation between luminal A and basal subtypes results in distinct clustering when looking at MMTR expression (Fig. 8).

It is important to note that drugs identified in Fig. 7 were predicted to have better efficacy when the CAC is dysregulated, yet they are not thought to directly target the CAC. Though they were identified specifically on the dysregulated CAC gene signature, the predictions do not explain whether CAC dysregulation is mechanistically responsible for the increased efficacy, or if CAC dysregulation co-occurs with another feature that is mechanistically responsible. These findings are hypothesis generating, not conclusive. In order to address this, one would need mechanistic insights into why a wee-1 inhibitor (681640) and an anti-folate (methotrexate), for example, are more effective in cell types with dysregulated CAC and then experimentally manipulate the CAC, or the hypothesized co-occurring mechanism, to ask if this alters their efficacy.

An important caveat to this type of analysis is the limitations of how far transcriptomic data can be equated to metabolic dysregulation, which occurs to a great extent at the post-transcriptional and post-translational levels. Thus, interpretation of results from such analysis, while hypothesis generating, needs to be followed up with proteomic and metabolomic data, to fully investigate predicted metabolic weaknesses for therapeutic exploitation. To better understand metabolic flux across types of cancer and even among subtypes within a particular cancer type, more unbiased metabolomics studies need to be conducted to fully appreciate the role of metabolic reprogramming in cancer initiation, progression, and prognosis^59^. Despite such limitation, this transcriptome-based approach provides insights that can drive more focused lines of research incorporating targeted metabolomics and proteomics studies. The high level of correlation observed between studies in separate cohorts of patients with the same disease combined with the fact that mapping of metabolic circuits predicts changes in metabolites that have been previously published provide confidence that this method is a highly informative for novel insights. This analytical pipeline can be applied to any transcriptional data to infer patterns of metabolic reprogramming, in any disease setting.

## Methods

### Pan-cancer DEG analysis

The results published here are in whole based upon data generated by TCGA^12^ Research Network (http://cancergenome.nih.gov/). Firehose, a web portal site that has been developed by the Broad Institute, (https://gdac.broadinstitute.org) aiming to deliver automated analyses of the TCGA data to general users, was utilized to download the preprocessed, Level 3, RSEM transcriptomic data. Gene expression data was analyzed using Bioconductor 3.1 (http://bioconductor.org), running on R 3.1.3. RNA-sequencing RSEM counts were processed to remove genes lacking expression in more than 80% of samples. To identify DEGs, primary tumor samples (samples ending in “0.01”) were compared to their matched normal tissues (samples ending in “0.11”), in their respective tissues. Scale normalization and moderated Student's *t* tests were performed using empirical Bayes statistics in the “Limma”^60^ package. The resulting *p* values were adjusted for multiple testing using the false discovery rate Benjamini and Hochberg correction method (Supplementary Data 7).

### Code availability

Code is available upon request to the corresponding author.

### Pathway score

Gene and pathway scores were calculated in R 3.1.3. DEG lists for each cancer site were used to assign individual gene scores. Gene scores (Eq. 1) were designated by taking the absolute value of the log FC multiplied by the –log(adjusted *p* value):

1 $${\mathrm{Gene}}\;{\mathrm{score = }}\left| {{{\log\,FC}}^ \ast {{ - \log}}\left( {{\mathrm{adj}}.p.{\mathrm{val}}} \right)} \right|.$$

Metabolic pathways were then downloaded from the KEGG^35^ were downloaded. Genes from each of the 114 pathways are reported in Supplementary Data 2. Pathway scores (Eq. 2) were then calculated by summing the gene scores for all genes within each of the pathways and dividing by the square root of the sample size for that particular tissue, to account for sample size effects in different cancer sites:

2 $${\mathrm{Pathway}}\;{\mathrm{score = \Sigma }}\left( {{\mathrm{Gene}}\;{\mathrm{scores}}} \right){\mathrm{/}}\surd {\mathrm{n}}.$$

All pathway scores were then exported into a table, to determine statistical significance of each score (Supplementary Data 3). Pathways were clustered into 10 major categories based upon KEGG classifications.

### Bootstrapping for pathway score statistical significance

Bootstrapping^61^ is a technique based on random sampling with replacement. Using R 3.1.3, pathway scores were randomly generated 100,000 times per pathway, based on the number of genes in the pathway, and plotted into a distribution. The scores for each of those pathways were then plotted against the distribution and a *p* value was calculated based on where that score lies within the distribution of scores (Supplementary Figure 5). Using all *p* values, the pathway score table (Supplementary Data 4) was adjusted to only include those scores that were considered to be statistically significant. All other values were replaced with “0” (Supplementary Data 5).

### Pathway scores heatmap

Bootstrapped pathway scores were utilized to create pathway score heatmaps in R 3.1.3, constructed using the “Gplots” and “Heatmap.2” packages in R. Data were scaled and Euclidian distances and hierarchical clustering were applied using the “h.clust” function. All 0 values (non-significant pathway scores) are represented as gray. For specific pathway heatmaps, at the gene level, fold-change values from the initial Limma output for each cancer type was utilized. Data were scaled using a min to max calculation (Eq. 3):

3 $$\left( m{{\mathrm{ - min}}\left( m \right)} \right){\mathrm{/}}\left( {{\mathrm{max}}\left( m \right){\mathrm{ - min}}\left( m \right)} \right).$$

Once again Euclidian distances and hierarchical clustering was applied. Heatmaps of the significantly DEGs are represented by blue (negative) or red (positive), and non-significantly DEGs are represented as gray (Supplementary Data 8 and 9).

### Pathway maps

Pathway maps were generated using the Cytoscape^62^ software, and specifically the VizMapper functions. Pathway maps were based on existing pathway maps in KEGG^35^. Limma output for DEG analysis was utilized to direct shading of genes within the pathway: red (positive fold change, statistically significant), blue (negative fold change, statistically significant), or gray (non-statistically significant), for individual cancer sites.

### Dose–response cell viability

PC-3 and DU145 cells were obtained from ATCC (Manassas, CA, USA). MDA-MB-231 cells were provided by Dr. John Ebos, Ph.D. (Department of Cancer Genetics, Genomics and Development, Roswell Park Comprehensive Cancer Center (RPCCC), Buffalo, NY, USA). 786-O and ACHN cells were provided by Dr. Eric Kauffman, M.D. (Department of Medicine, Roswell Park Comprehensive Cancer Center (RPCCC), Buffalo, NY, USA). HS578T cells were provided by Dr. Mikhail Nikiforov, Ph.D. (Department of Cell Stress Biology, Roswell Park Comprehensive Cancer Center (RPCCC), Buffalo, NY, USA). MCF7 and T47D cells were provided by Dr. Katerina Gurova, M.D., Ph.D. (Department of Cell Stress Biology, Roswell Park Comprehensive Cancer Center (RPCCC), Buffalo, NY, USA). All cells were mycoplasma tested prior to use by respective labs and either ATCC certified or STR profiled. All prostate cancer cells (DU145 and PC-3) were maintained in RPMI-1640 medium supplemented with 10% fetal bovine serum (FBS) and 1% antibiotics. Other cells were maintained in Dulbecco's modified Eagle's medium with 10% FBS and 1% antibiotics. Metformin and sulfasalazine were obtained from Sigma and BENSpm was purchased from Synthesis Med Chem (Shanghai, China). Cells were seeded in 96-well plates at 3000 cells/well on day 0. They then underwent either 48 h (BENSpm and Sulfasalazine) or 72 h (metformin) of treatment. Resazurin (Sigma) was then added to each well and allowed to incubate for 2 h at 37 °C. The plates were then read on a spectrophotometer by excitation at 570 nm and reading of the fluorescence at 600 nm. Dose–response curves were then plotted using Prism GraphPad 7.

### MMTR analysis

In order to characterize regulatory networks, we used iRegulon^48^, a Java add-on in Cytoscape, to identify MMTRs. In this approach, we use a large collection of TF motifs (9713 motifs for 1191 TFs) and a large collection of ChIP-seq tracks (1120 tracks for 246 TFs). This method relies on a ranking-and-recovery system where all genes of the human genome (hg19) are scored by a motif discovery step integrating the clustering of binding sites within *cis*-regulatory modules (CRMs), the potential conservation of CRMs across 10 vertebrate genomes, and the potential distal location of CRMs upstream or downstream of the transcription start site (TSS ±10 kb). The recovery step calculates the TF enrichment for each set of genes, input for each of the individual analyses, leading to the prediction of the TFs and their putative direct target genes, which exist in the input lists. This method optimizes the association of TFs to motifs using both direct annotations and predictions of TF orthologs and motif similarity.

### MMTR correlation analysis

Correlation values between MMTRs and all expressed genes were derived in R 3.1.3, constructed using the “cor” function across all patients in both TCGA-PRAD and KICH cohorts. The empirical cumulative distribution function for each complete MMTR correlation profile (background) was determined via the “ecdf” function, and similarly for the MMTR correlation profiles against polyamine biosynthetic genes only. Significant shift in distributions between MMTR/background and MMTR/polyamine biosynthetic gene correlations was assessed by Kolmogorov–Smirnov test. (Supplementary Data 10)

### Common mutation analysis

Using cBioPortal (http://www.cbioportal.org), patients in the PRAD cohort were queried for either co-occurent relationships or mutually exclusive relationships between the list of most commonly occurring mutations in PRAD and the four MMTRs in question. CBioPortal is a publically available database, which based on all of the genomic data available for PRAD constructs a list of the most commonly occurring mutations. Additionally, it calculates the significance of co-occurrence or mutual exclusivity, based upon the Mutual Exclusivity Modules (MEMo)^63^. MEMo is a method that searches and identifies modules based upon: (1) genes recurrently altered across a set of tumor samples; (2) genes known to or likely to participate in the same biological process; and (3) alteration events within the modules are mutually exclusive. Using this information, it then integrates multiple data types and maps genomic alterations to biological pathways and uses a statistical model that predicts the number of alterations both per gene and per sample.

### GTF2B and ERG-binding site overlap

ChIP-seq BED files were downloaded from the Cistrome database (http://www.cistrome.org), corresponding with three different studies. To confirm overlap between GTF2B and ERG: K562 erythroblast; bone marrow untreated from the Martens et al.^64^ study and GTF2B K562 erythroblast; bone marrow untreated from the Pope et al.^65^ study were downloaded. GenomicRange was then used to determine the overlap between these peaks in the same line. Then, to determine ERG peaks in polyamine biosynthetic genes in prostate cancer cell lines specifically, the VCaP; epithelium; prostate ERG-non-treated data from Sharma et al.^66^ was downloaded and imported into the Interactive Genome Viewer (https://software.broadinstitute.org/software/igv/) to visualize peaks in these genes. GTF2B ChIP-seq data was not available for GTF2B in prostate cancer.

### PAM50 BRCA analysis

The PAM50 is a method that has been previously described in the literature^50,51^. The PAM50 classification of tumors within the TCGA cohort was obtained^51^. This classification was then used to stratify patients into four major groups: basal-like, HER2-expressing, luminal A, and luminal B. Any patients with no classification within this file were removed from the analyses and all data were preprocessed as outlined in the Pan-cancer DEG analysis section. Post-normalization, all tumors still included underwent unsupervised clustering based on the expression of all metabolic genes within the cohort. Additionally, comparisons of patients within each cohort were then made with normal tissues to obtain Pathway Scores (see Pan-cancer DEG analysis section) for each of the patient cohorts (Supplementary Data 11). MMTR analysis was then performed to determine MMTRs of uniquely dysregulated pathways within the basal-like and luminal A subtypes. Patients from each of those subtypes then underwent unsupervised hierarchical clustering based on the gene expression of those MMTRs of uniquely dysregulated pathways.

### BRCA cell line analysis

RNA-sequencing data was obtained for 28 different normal breast, luminal breast cancer, and basal breast cancer cell lines^56^. All data were preprocessed as outlined in the Pan-cancer DEG analysis section. Post-normalization, all tumors still included underwent unsupervised clustering based on the expression of all metabolic genes within the cohort. Additionally, comparisons of the cell lines from each of those subtypes then underwent unsupervised hierarchical clustering based on the gene expression of those MMTRs of uniquely dysregulated pathways, from the patient data.

### Drug screening data

The Genomics of Drug Sensitivity in Cancer database^54^ (https://www.cancerrxgene.org) was accessed to download detailed IC_50_ data for phenformin in breast cancer cell lines. Cell lines were then split into luminal and basal based on ATCC and literature searches. Those cell lines regarded as HER2+ were excluded. Mann–Whitney *U* -test was then applied to determine significance. DeSigN^55^ (http://design.cancerresearch.my) was then used to determine a drug that would target the CAC in an unbiased manner. Based on the hierarchical clustering of TCGA patients based on the expression levels (fold change over normal), two distinct clusters of cancer were determined, those that largely up-regulate the genes within the pathway and those that down-regulate the genes. The signature (up-regulated and down-regulated genes) was then input into DeSigN^55^ to identify a drug of interest (Fig. 7d). The IC_50_ data for this drug was then downloaded for all cell lines and analyzed for differences in sensitivity between the two CAC expression groups, and luminal and basal breast cancer cell lines. Mann–Whitney *U* tests were utilized to determine if there were significant differences between basal and luminal cell line data.

## Electronic supplementary material

Supplementary Information

Description of Additional Supplementary Files

Supplementary Dataset 1

Supplementary Dataset 2

Supplementary Dataset 3

Supplementary Dataset 4

Supplementary Dataset 5

Supplementary Dataset 6

Supplementary Dataset 7

Supplementary Dataset 8

Supplementary Dataset 9

Supplementary Dataset 10

Supplementary Dataset 11

Source Code File

Life Sciences Checklist

## Data Availability

All TCGA data are available for download through Firehose, a web portal site that has been developed by the Broad Institute (https://gdac.broadinstitute.org), aiming to deliver automated analyses of the TCGA data to general users. Microarray data can be downloaded from the NCBI Gene Expression Omnibus for prostate adenocarcinoma (GSE21032), lung adenocarcinoma (GSE2514,), and breast adenocarcinoma (GDS3324). All cell line drug data can be downloaded from the Sanger Genomics of Drug Sensitivity in Cancer database (https://www.cancerrxgene.org).
