# Supplementary material for: Pan-cancer analysis of transcriptional metabolic dysregulation using The Cancer Genome Atlas
Source: Nat Commun. 2018 Dec 14;9:5330. doi: 10.1038/s41467-018-07232-8 (PMC6294258; doi:10.1038/s41467-018-07232-8)
Supplement: Supplementary file 1 — Supplementary Information [file 41467_2018_7232_MOESM1_ESM.pdf]

Supplementary Figure 1. Confirmation of Pathway Scores in Alternate Data

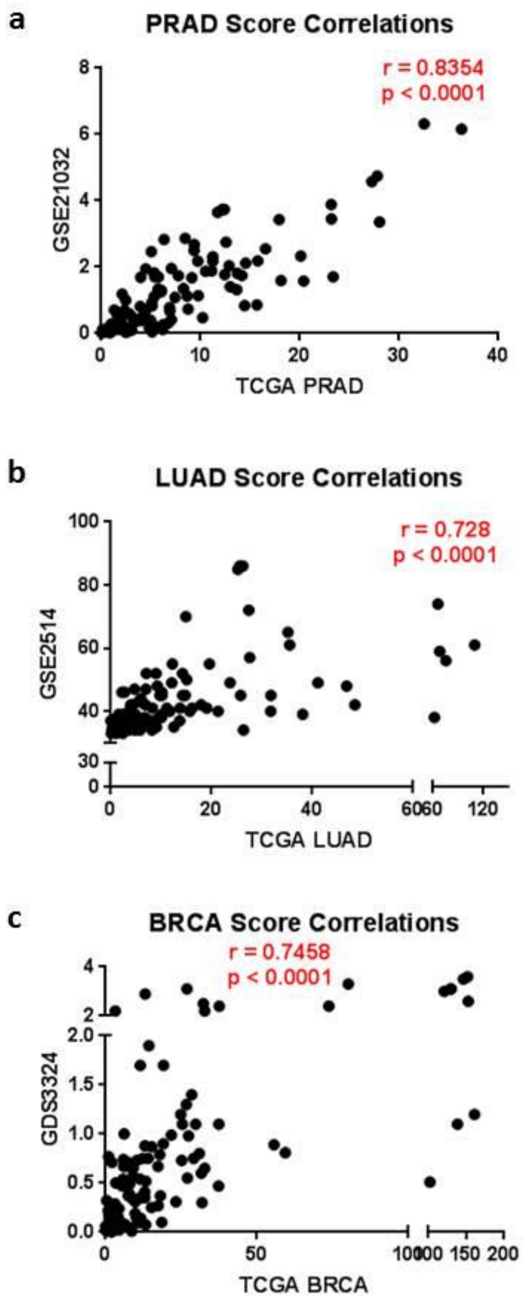

Supplementary Figure 1. Highly Significant Correlation of Metabolic Pathway with TCGA RNA-sequencing data and Microarray Prostate, Lung and Breast Data (A) There is a highly statistically significant positive correlation between the Taylor, et al. (2010) radical prostatectomy metabolic scores and the TCGA radical prostatectomy scores. (B) There is a highly statistically significant positive correlation between the Stearman RS, et al. (2005) lung adenocarcinoma and TCGA lung adenocarcinoma scores. (C) There is a highly statistically significant positive correlation between the Casey T, et al. (2009) breast carcinoma and TCGA breast carcinoma scores.

Supplementary Figure 2. Pentose Glucuronate Interconversion in Liver Hepatocellular Carcinoma

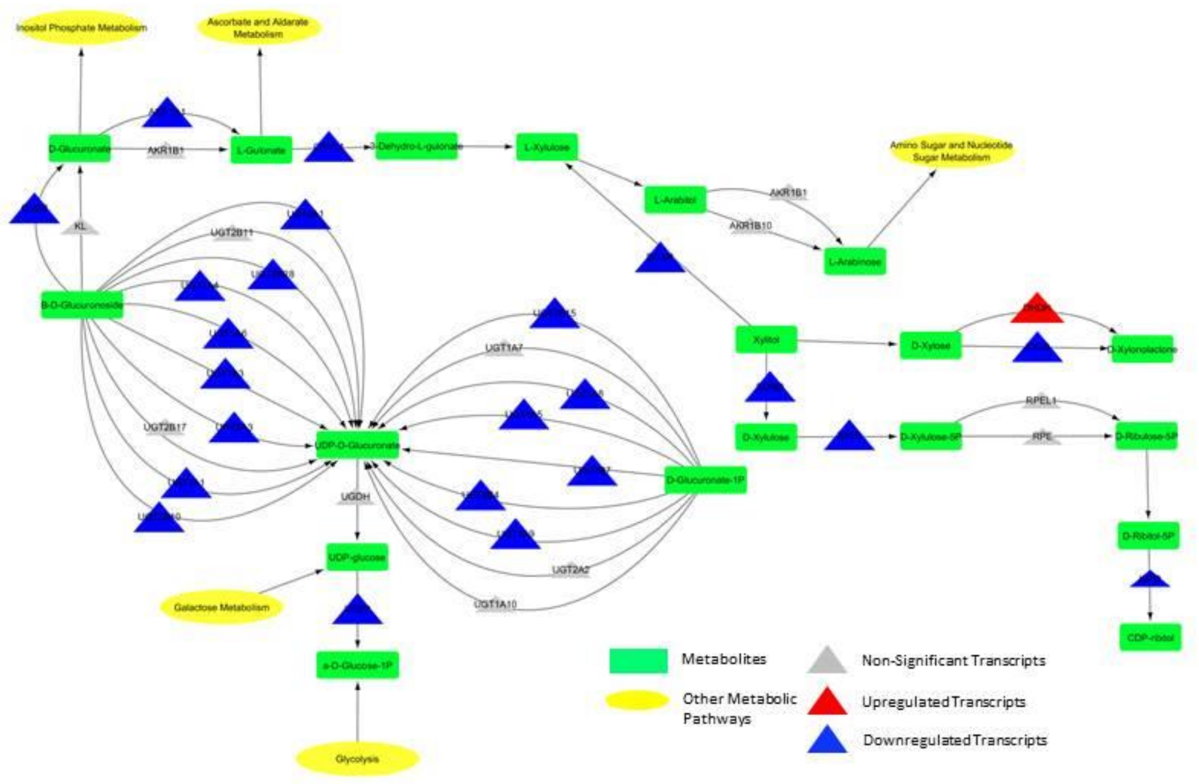

Supplementary Figure 2. Model of the Pentose Glucuronate Interconversion Pathway in LIHC, which has a large number of statistically significant down-regulated genes. Pathway displays genes (triangles) shaded by direction (red and blue, up and down, respectively) and significance and sized by fold change differences. Pathways also include metabolite outputs (green rectangles) and connected pathways (yellow ellipse).

Supplementary Figure 3. Polyamine Biosynthetic Pathway in Kidney Chromophobe

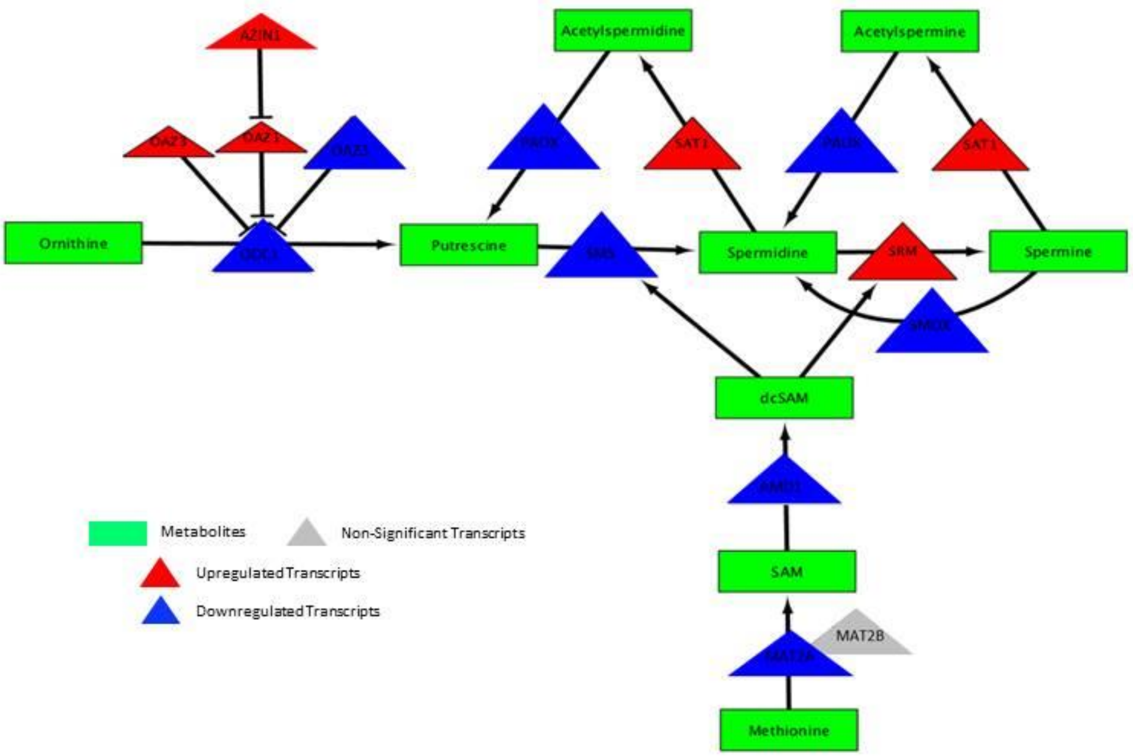

Supplementary Figure 3. Model of the Polyamine Biosynthetic Pathway in KICH, which has a large number of statistically significant down-regulated genes. Pathway displays genes (triangles) shaded by direction (red and blue, up and down, respectively) and significance and sized by fold change

differences. Pathways also include metabolite outputs (green rectangles).

Supplementary Figure 4. Prostate Cancer Polyamine Biosynthesis Master Regulators and Polyamine gene distributions

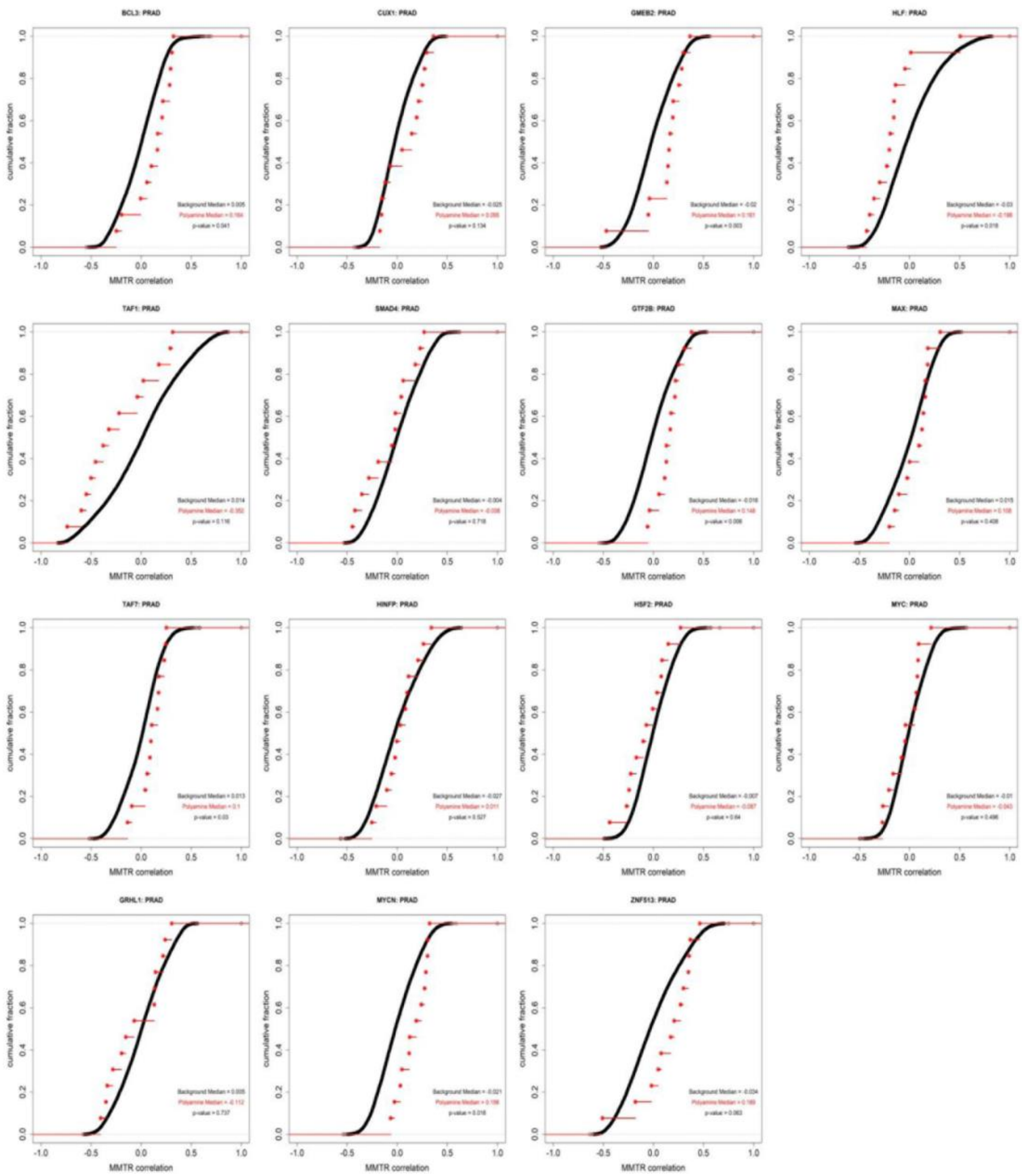

Supplementary Figure 4. Prostate Cancer Polyamine Biosynthesis Master Regulators and Polyamine gene distributions. Prostate cancer cumulative distribution frequencies showing MMTR correlation with every gene in the genome, with red dots indicating the correlations with Polyamine Biosynthetic genes, show a distinct pattern and high level of statistically significant correlation values between MMTRs and Polyamine Biosynthetic genes. P-values for all MMTRs and Polyamine genes are reported in the table, with significant associations highlighted in red

Supplementary Figure 5. Kidney Chromophobe Polyamine Biosynthesis Master Regulators and Polyamine gene distributions

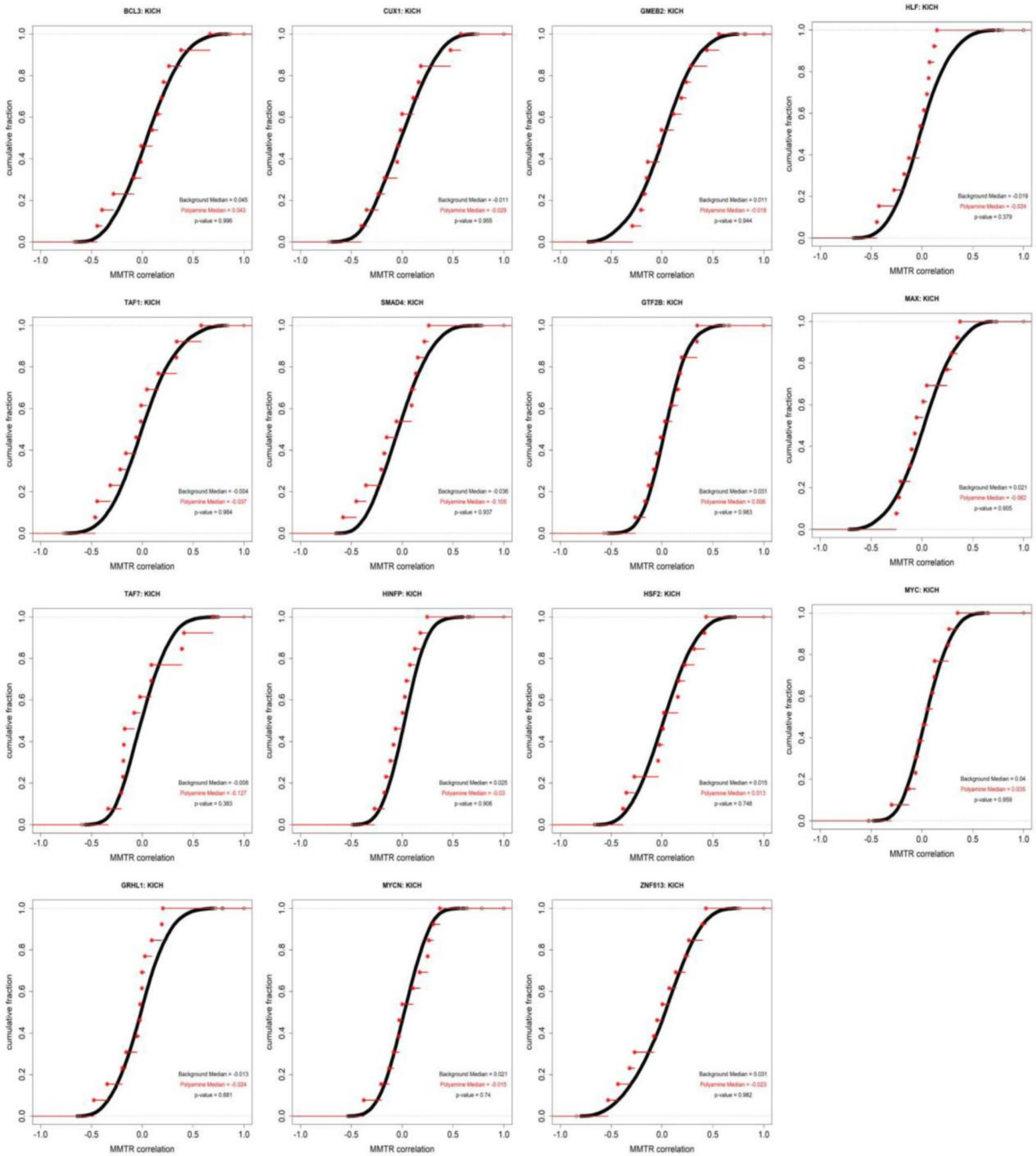

Supplementary Figure 5. Kidney Chromophobe Polyamine Biosynthesis Master Regulators and Polyamine gene distributions. Kidney chromophobe cumulative distribution frequencies showing MMTR correlation with every gene in the genome, with red dots indicating the correlations with Polyamine Biosynthetic genes, show no distinct pattern and a little statistically significant correlation values between MMTRs and Polyamine Biosynthetic genes. P-values for all MMTRs and Polyamine genes are reported in the table, with significant associations highlighted in red

Supplementary Figure 6. GTF2B and ERG ChIP-seq Peak Overlap

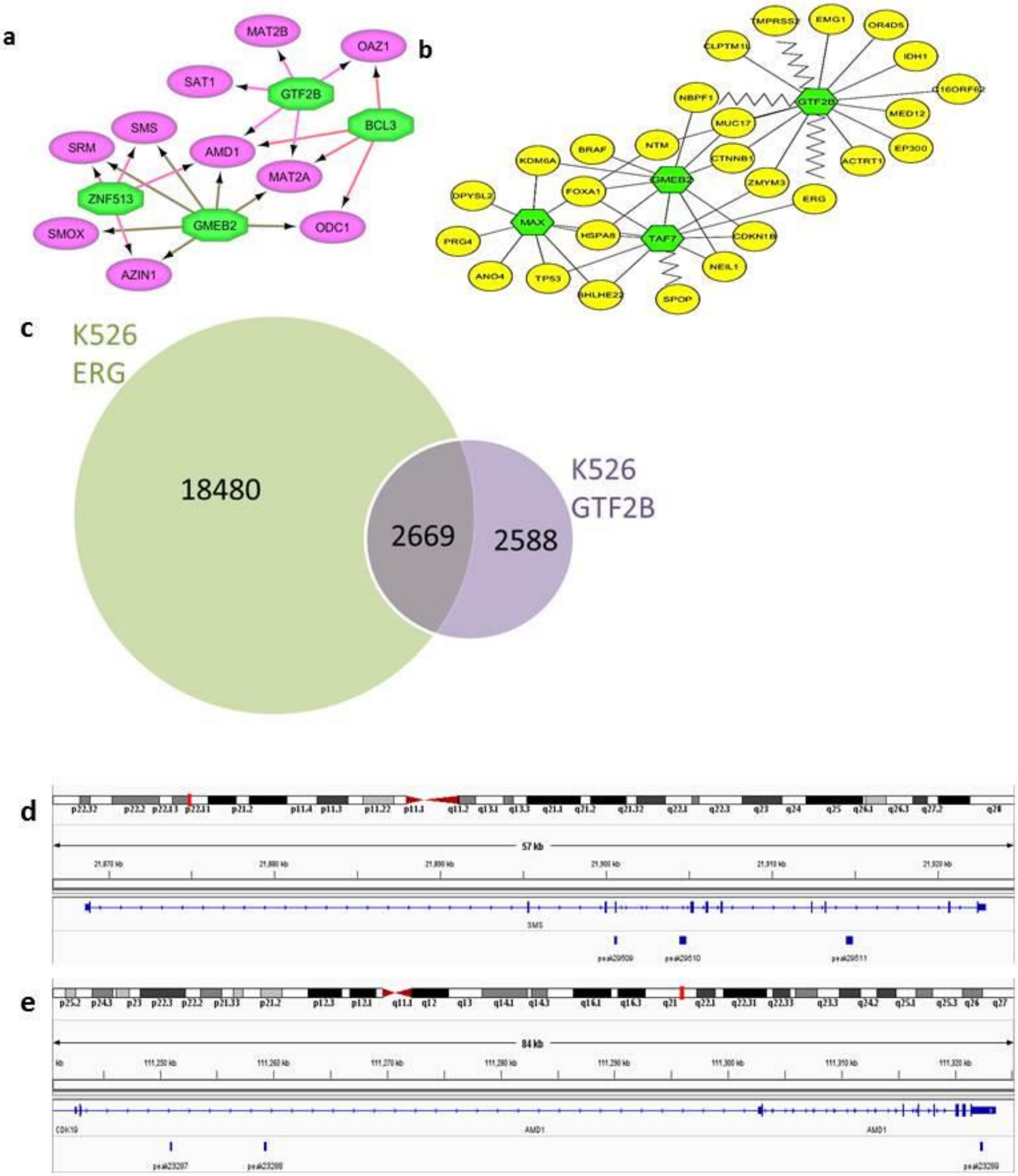

Supplementary Figure 6. Polyamine Biosynthesis Master Regulators and Overlap of GTF2B and ERG genes. (A) Network of top 4 Master Transcription Factors (green) and the Polyamine Biosynthetic Genes (purple) affected by those transcription factors. (B) Network of Master Transcription Factors (green) and commonly mutated genes in PRAD (yellow) indicating gene co-occurrence (straight edges) or mutual exclusivity (zig-zag edges), (C) ChIP-sequencing tracks downloaded from Cistrome, in K562 Hematopoietic cells the number of overlapping peaks were calculated. (D) Examples of ERG peaks using Interactive Genome Viewer (IGV) in polyamine genes from VCaP cells for SMS (E) and AMD1 (bottom).

Supplementary Figure 7. Bootstrapping Example

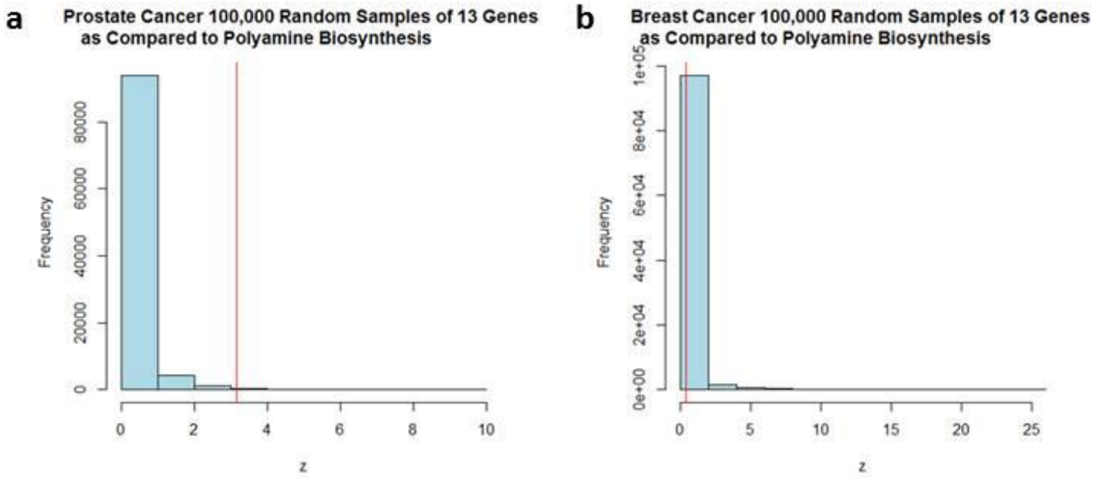

Supplementary Figure 7. Examples of statistically significant and non-statistically significant bootstrapping plots (A) When randomly sampled 100,000 times, the Breast Cancer Score (red line) for the 13 genes in the Polyamine Biosynthetic Pathway sits within the distribution and is not considered statistically significant by bootstrapping (B) When randomly sampled 100,000 times, the Prostate Cancer Score (red line) for the 13 genes in the Polyamine Biosynthetic Pathway sits within the distribution and is not considered statistically significant by bootstrapping.
