# Supplementary material for: Pan-cancer analysis of transcriptional metabolic dysregulation using The Cancer Genome Atlas
Source: Nat Commun. 2018 Dec 14;9:5330. doi: 10.1038/s41467-018-07232-8 (PMC6294258; doi:10.1038/s41467-018-07232-8)
Supplement: Supplementary file 2 — Description of Additional Supplementary Files [file 41467_2018_7232_MOESM2_ESM.pdf]

Description of Additional Supplementary Files

File Name: Supplementary Data 1
Description: TCGA Sample Sizes

File Name: Supplementary Data 2
Description: Pathway gene lists

File Name: Supplementary Data 3
Description: TCGA Pathway Scores

File Name: Supplementary Data 4
Description: Bootstrapped TCGA Pathway Scores

File Name: Supplementary Data 5
Description: Bootstrapped TCGA Pathway Scores with Zeroes

File Name: Supplementary Data 6
Description: Defines how many cancers each pathway is statistically significantly altered in

File Name: Supplementary Data 7
Description: A compilation of differentially expressed gene lists for all TCGA disease sites. Heatmaps throughout the paper were constructed using the tables reported in Supplementary Data 8-10

File Name: Supplementary Data 8
Description: Provides the data frames for the Carbohydrate, Amino Acid, and Lipid pathway scores across all types of cancer

File Name: Supplementary Data 9
Description: Provides the data frames for the Pentose Glucuronate Interconversion and Polyamine Pathways across all types of cancer

File Name: Supplementary Data 10
Description: Provides the data frames for the PRAD and KICH Polyamine Master Regulator Correlations

File Name: Supplementary Data 11
Description: Bootstrapped Pathway Scores for the 114 metabolic pathways across the 4 Breast Cancer Molecular Subtypes
